# Effectiveness of thiazide diuretics in patients with hypertension: a systematic review and meta-analysis

**DOI:** 10.1097/MS9.0000000000004668

**Published:** 2026-01-06

**Authors:** Taimoor Ashraf, Fnu Chandni, Fnu Nancy, Nisha Devi, Deepa Bai, Ramsha Waseem, Maheen Jabbar, Muskan Turesh, Shah Dev, Rabia Bint I Zafar, Sadia Manan, Fatima Jabbar, Iqra Maryam, Zara Naveed, Sayed Jawad

**Affiliations:** a Department of Medicine, Nishtar Medical University, Multan, Pakistan; b Department of Medicine, Isra University, Hyderabad, Pakistan; c Department of Medicine, United Medical and Dental College, Karachi, Pakistan; d Department of Medicine, Bahria University of Medical and Dental College, Karachi, Pakistan; e Department of Internal Medicine, Peoples University of Medical and Health Sciences, Benazirabad, Pakistan; f Department of Medicine, Institute Shaheed Mohtarma Medical College Lyari, Karachi, Pakistan; g Department of Medicine, Bahria University Health Sciences, Karachi, Pakistan; h Department of Medicine, Ghulam Muhammad Mahar Medical College, Sukkur, Pakistan; i Nishtar Medical University, Multan, Pakistan; j Department of Medicine, Ziauddin Medical University, Karachi, Pakistan; k Department of Medicine, Jinnah Medical University, Karachi, Pakistan; l Department of Medicine, Dow University of Health Sciences, Karachi, Pakistan; m Department of Medicine, Kabul Medical University

**Keywords:** chlorthalidone, hydrochlorothiazide, hypertension, thiazide diuretics

## Abstract

**Background:**

Thiazide diuretics are commonly prescribed for the treatment of hypertension (HTN). However, the comparative efficacy and safety of hydrochlorothiazide (HCTZ) versus chlorthalidone (CTD) in managing patients with HTN remain unclear.

**Materials and Methods:**

A comprehensive literature search was performed using two electronic databases, MEDLINE and Cochrane, from their inception through November 2024. The aim was to identify randomized controlled trials or observational double-arm studies assessing the effects of HCTZ versus CTD on cardiovascular outcomes and safety in patients with HTN. Outcomes were reported as hazard ratios (HRs) with 95% confidence intervals (CIs), and analyses were conducted using a random effects model. A *P*-value of <0.05 was considered statistically significant.

**Results:**

Of the 409 articles identified in the initial search, six relevant studies were included in the final analysis. No significant differences were observed between the HCTZ and CTD arms for cardiovascular outcomes, including major adverse cardiovascular events, myocardial infarction, stroke, hospitalization for heart failure, and angina. However, patients receiving CTD experienced significantly higher rates of hypokalemia and hyponatremia compared to those on HCTZ. The risk of acute kidney injury did not differ significantly between the two groups.

**Conclusion:**

HCTZ and CTD showed comparable efficacy in terms of cardiovascular outcomes. Nevertheless, HCTZ appeared to have a safer profile, with a lower incidence of electrolyte imbalances, such as hypokalemia and hyponatremia, compared to CTD.

## Introduction

The global prevalence of hypertension (HTN) is increasing[1]. Worldwide, 8.5 million deaths caused by stroke, ischemic heart disease, vascular diseases, and renal disorders can be attributed to HTN, pre- HTN, and other dangerously high blood pressure conditions^[2,3]^. The 2017 American College of Cardiology/American Heart Association guidelines recommend thiazide and thiazide like diuretics as the first line treatment for HTN requiring pharmacological therapy[4].

HIGHLIGHTS

- Six studies were meta-analyzed
- No significant differences were noted for all the cardiovascular outcomes namely major adverse cardiovascular events, myocardial infarction, stroke, hospitalization for heart failure, and angina between the hydrochlorothiazide and chlorthalidone (CTD) arms.
- CTD was associated with significantly higher rates of hypokalemia and hyponatremia as compared

While head-to-head comparisons of these two first-line antihypertensive agents have been done, recently accumulating evidence suggests that chlorthalidone may be superior to HCTZ in terms of cardiovascular efficacy^[5,6]^. A recent network meta-analysis of randomized clinical trials (RCTs) also suggested that chlorthalidone (CTD) is significantly more effective than hydrochlorothiazide (HCTZ) in reducing the risk of major adverse cardiovascular events (MACE)[7]. However, this meta-analysis did not evaluate the individual components of MACE.

Moreover, the safety profile, specifically with respect to electrolyte disturbances and acute kidney injury (AKI), has not been extensively explored either. Therefore, given conflicting reports and the recent publication of additional evidence^[8,9]^, we conducted a meta-analysis aiming to ascertain the cardiovascular efficacy and safety of HCTZ versus CTD. The primary objective of this systematic review and meta-analysis was to compare the cardiovascular efficacy of HCTZ and CTD in patients with HTN. Specifically, we assessed their effects on MACE, myocardial infarction (MI), stroke, hospitalization for heart failure (HHF), and angina. The secondary objective was to evaluate and compare the safety profiles of the two agents, focusing on the incidence of hypokalemia, hyponatremia, and AKI. This systematic review adheres to the TITAN Guidelines 2025 for transparent and responsible use of AI technologies in research reporting and writing processes, where applicable[10].

## Methods

This systematic review and meta-analysis is registered with PROSPERO (ID: CRD42025632848). It has been reported in concordance with guidelines provided by the Preferred Reporting Items for Systematic Reviews and Meta-Analysis (PRISMA) statement and Assessing the methodological quality of systematic reviews (AMSTAR 2) guidelines. Approval from the institutional review board was not required since the data were publicly available.

### Literature search strategy and data sources

We systematically searched two databases (MEDLINE and Cochrane) for RCTs and/or double-arm observational studies assessing the impact HCTZ versus CTD in patients with HTN from inception through November 2024, without any time or language restrictions (Supplemental Digital Content Table 1, available at: http://links.lww.com/MS9/B71). Additional sources included bibliographies of review articles, original studies, and relevant editorials. Mesh terms along with Boolean operators were used to devise an effective search strategy for each database (Supplemental Digital Content Table 1, available at: http://links.lww.com/MS9/B71).

### Study selection

All articles retrieved from the systematic search were exported to EndNote Reference Manager (Version X7.5; Clarivate Analytics, Philadelphia, Pennsylvania) where duplicates were sought and removed. The remaining articles were then assessed at title and abstract level by two investigators (DB and ND), after which full text were read to confirm relevance. Any disagreements were resolved by mutual discussion with a third investigator (TA). The following pre-defined inclusion criteria was used: (1) studies which investigated the effects of HCTZ versus CTD in patients with HTN (2) were RCTs and/or double-arm observational studies, and (3) evaluated cardiovascular and safety outcomes. Studies comparing HCTZ and CTD as a part of a combination regimen with other medications were excluded.

### Data extraction and outcomes of interest

Two investigators (DB and ND) autonomously extracted data from the selected studies on pre-specified collection forms. Apart from baseline trial characteristics, data were abstracted for cardiovascular outcomes namely MACE, MI, stroke, HHF, and angina. In addition, data were extracted for safety outcomes as well namely hypokalemia, hyponatremia, and AKI. The potential risk of bias of the short-listed studies was evaluated using the modified Cochrane Collaboration’s risk of bias tool and Newcastle–Ottawa Scale.

### Statistical analyses

All statistical analyses were performed using Review Manager (version 5.4.1). Evaluations were reported as hazard ratios (HRs) with 95% confidence intervals (CIs). Logit transformation for HRs was carried out and meta-analysis was done using a generic inverse variance random-effects model. In a few cases where the HR was not available for a MACE outcome, raw event rates were used to calculate its risk ratios (RRs) which were then pooled together with the HRs. Inclusion of a limited number of studies did not permit the evaluation of a publication bias. Heterogeneity was assessed using the Higgins I^2^ statistic, with an I^2^ > 50% being considered significant heterogeneity. In cases of significant heterogeneity, a sensitivity analysis was performed by removing one study at a time. A *P*-value <0.05 was considered statistically significant in all cases.

## Results

### Literature search

**Figure 1.**
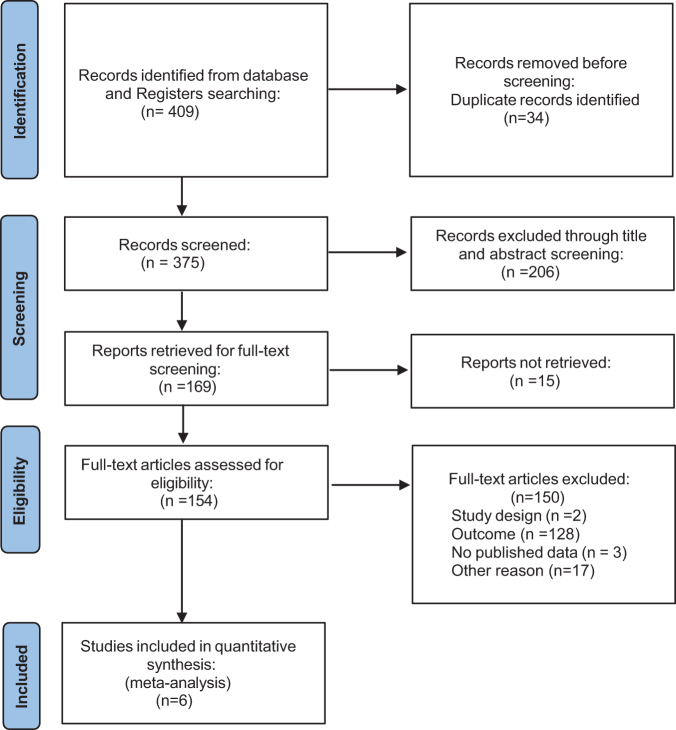
PRISMA flowchart for literature search strategy. PRISMA, Preferred Reporting Items for Systematic Reviews and Meta-Analysis.

The initial literature search yielded 409 potentially relevant articles. After applying our predetermined eligibility criterion, six studies were selected for inclusion in this meta- analysis^[5,8,9,11–13]^. The PRISMA flowchart (Fig. 1) demonstrates the detailed search and study selection process.

### Study characteristics and risk of bias assessment

A total of 774 488 patients (74% males; mean age, 49.6 years) with a median follow-up of 16.7 months were included in our meta-analysis (Table 1).

**Table 1:** Characteristics of included trials

Trial characteristics	Dhalla *et al.*		Hripcsack *et al.*		Neutel *et al.*		Ishani *et al.*		Edwards *et al.*		Dorsch *et al.*	
	CTD	HCTZ	CTD	HCTZ	CTD	HCTZ	CTD	HCTZ	CTD	HCTZ	CTD	HCTZ
Sample size (n)	10 384	19 489	36 918	693 337	418	419	6756	6767	2936	9786	2392	4049
Mean age (SD)	73 (6)	73.0 (5.9)	49.0 (10 · 4)	48.2 (10.6)	58.5 (10.8)	57.6 (10.8)	72.4 (5.4)	72.5 (5.3)	74(7)	74(7)	46.9(5.9)	46.7(5.7)
Median follow-up duration (months)	8.5	13.3	N/A	N/A	0.5	0.5	28.8	28.8	21.6	24.3	7	7
Females (%)	6160 (59.3)	11 505 (59)	7310 (51.8)	175 600 (61.1)	192 (46)	171 (41)	220 (3.3)	211 (3.1)	1599 (54)	4322 (44)	-	-
Medical history												
Mean SBP (SD)	N/A	N/A	N/A	N/A	168.2 (7.1)	167.6 (7.0)	139 (14)	139 (14)	N/A	N/A	142.1 (13.7)	142.7 (13.1)
Mean DBP (SD)	N/A	N/A	N/A	N/A	95.7 (9.2)	95.7 (9.6)	N/A	N/A	N/A	N/A	N/A	N/A
Diabetes mellitus (%)	1289 (12)	2691 (14)	630 (4.5)	13 200 (4.6)	62 (15)	58 (14)	2967 (43.9)	3062 (45.2)	1322(45)	4122(42)	125 (3.1)	77(3.2)
Mean eGFR<60 ml/min/1.73 m2 (%)	N/A	N/A	N/A	N/A	54 (13)	41 (10)	1550 (22.9)	1547 (22.9)	914(32)	2491(26)	N/A	N/A
CKD (%)	22 (0.2)	42 (0.2)	N/A	N/A	58 (14)	50 (12)	N/A	N/A	N/A	N/A	N/A	N/A

CKD, chronic kidney disease; CTD, chlorthalidone; DBP, diastolic blood pressure; eGFR, estimated glomerular filtration rate; HCTZ, hydrochlorothiazide; NA, Not Available; SBP, systolic blood pressure; SD, standard deviation.

### Cardiovascular outcomes

#### MACE

**Figure 2.**
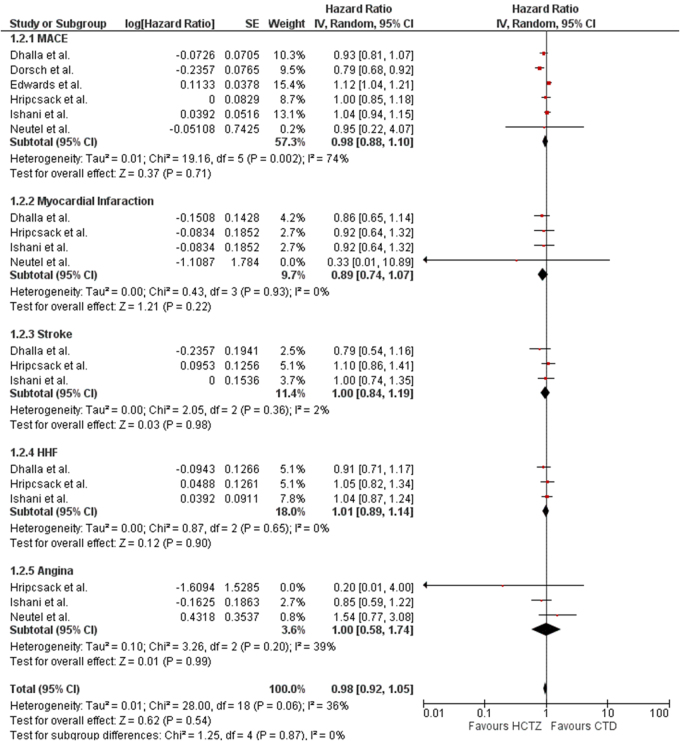
Forest plot evaluating cardiovascular outcomes (MACE, myocardial infarction, stroke, HHF and angina) between patients receiving HCTZ versus CTD. CTD, chlorthalidone; HCTZ, hydrochlorothiazide; MACE, major adverse cardiovascular events.

This outcome was reported by four studies (Fig. 2). The overall effect of HCTZ in reducing occurrences of MACE was similar to CTD (HR: 0.98; 95% CI: 0.88–1.10; *P* = 0.71; I^2^ = 74%).

#### MI

This outcome was reported by four studies (Fig. 2). The effect of HCTZ in reducing occurrences of MI was similar to CTD (HR: 0.94; 95% CI: 0.80–1.11; *P* = 0.46; I^2^ = 0%).

#### Stroke

This outcome was reported by three studies (Fig. 2). The effect of HCTZ in reducing the incidence of stroke was similar to CTD (HR: 1.00; 95% CI: 0.84–1.19; *P* = 0.98; I^2^ = 2%).

#### HHF

This outcome was reported by three studies (Fig. 2). The overall effect of HCTZ in reducing the chances of HHF was similar to CTD (HR: 1.01; 95% CI: 0.89–1.14; *P* = 0.90; I^2^ = 0%).

#### Angina

This outcome was reported by three studies (Fig. 2). The effect of HCTZ in reducing the occurrence of angina was similar to CTD (HR: 1.00; 95% CI: 0.58–1.74; *P* = 0.99; I^2^ = 39%).

### Safety outcomes

#### Hypokalemia

**Figure 3.**
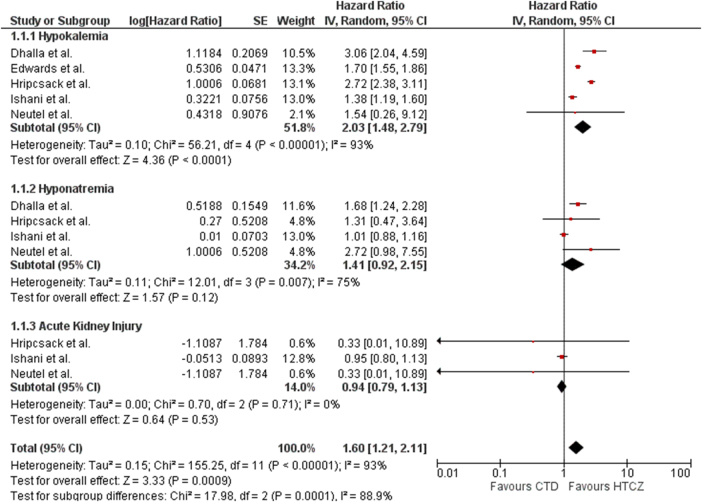
Forest plot evaluating safety outcomes (hypokalemia, hyponatremia, acute kidney injury) between patients receiving HCTZ versus CTD. CTD, chlorthalidone; HCTZ, hydrochlorothiazide.

This outcome was reported by four studies (Fig. 3). The effect of HCTZ in reducing the incidence of hypokalemia was significantly greater than that of CTD (HR: 2.03; 95% CI: 1.48–2.79; p < 0.0001; I^2^ = 93%). On performing sensitivity analysis, removing the study by Ishani et al. reduced the heterogeneity while the results remained consistent (HR: 2.32; 95% CI: 1.59–3.38; *P* < 0.00001; I^2^ = 0%).

#### Hyponatremia

This outcome was reported by four studies (Fig. 3). The effect of HCTZ in reducing the incidence of hypokalemia was significantly greater than that of CTD (HR: 1.32; 95% CI: 1.03–1.70; *P* = 0.03; I^2^ = 80%). On performing sensitivity analysis, removing the study by Ishani *et al* reduced the heterogeneity while the results remained consistent (HR: 1.49; 95% CI: 1.15–1.93; *P* = 0.002; I^2^ = 50%).

#### AKI

This outcome was reported by three studies (Fig. 3). The effect of HCTZ in reducing the incidence of AKI was similar to CTD (HR: 1.00; 95% CI: 0.84–1.19; *P* = 0.98; I^2^ = 84%). On performing sensitivity analysis, removing the study by Ishani *et al* HCTZ significantly decreased the occurrence of AKI as compared to CTD (HR: 1.37; 95% CI: 1.15–1.63; *P* = 0.0005; I^2^ = 0%), while eliminating heterogeneity as well.

### Risk of bias assessment

The two included observational studies were judged to have a low risk of bias based on their design, adjustment for confounders, and completeness of follow-up (Supplemental Digital Content Table 2, available at: http://links.lww.com/MS9/B71). However, the two randomized controlled trials had domains with unclear risk of bias, particularly regarding allocation concealment and blinding of outcome assessment. While both RCTs reported adequate randomization methods, the lack of detailed reporting on other methodological aspects limited our ability to fully assess their internal validity. Overall, the risk of bias was considered low for the observational studies and unclear for the randomized trials (Supplemental Digital Content Figure 1, available at: http://links.lww.com/MS9/B71, and Supplemental Digital Content Figure 2, available at: http://links.lww.com/MS9/B71).

## Discussion

This meta-analysis including over 770 000 patients compared the efficacy and safety of chlorthalidone and HCTZ in patients with HTN and yielded several key findings. Pooled results from our analysis revealed that both pharmacological therapies exhibit similar efficacy in terms of cardiovascular outcomes, namely MACE, MI, HHF, stroke, and angina. However, a notable distinction emerged in terms of the safety profiles of the two diuretics whereby HCTZ was significantly more effective in reducing the incidence of hypokalemia and hyponatremia.

Our findings conflict with those of the most recent network meta-analysis, which demonstrated that CTD was significantly more effective in reducing the risk of MACE as compared to HCTZ (HR: 0.79; 95% CI: 0.72–0.88; *P* < 0.0001)[14]. Although our findings differ from those of a recent network meta-analysis that reported superior efficacy of chlorthalidone over HCTZ for reducing MACE, this discrepancy may be attributable to differences in study selection and the incorporation of more recent evidence in our analysis. Given the pharmacologic similarities between the two agents, including shared mechanisms of action, it is plausible that their effects on cardiovascular outcomes may converge when evaluated in a more contemporary and comprehensive evidence base.

While CTD depicted similar cardiovascular efficacy to HCTZ, it is to be noted that most trials evaluating the efficacy of HCTZ are reported to utilize relatively higher doses of HCTZ compared with the other drugs used[15]. Whether or not the dosage of these thiazide diuretics impacts disease progression and cardiovascular outcome modification in patients with HTN remains unclear. This indicates a pertinent need for future research to conduct dose adjusted analysis comparing these two pharmacological regimens.

Meanwhile, our findings with respect to hypokalemia are in adherence with those of a recent meta-analysis[7]. However, safety profiles of the two diuretics otherwise have not been extensively evaluated and the observed disparity in safety outcomes might be attributed to differences in their pharmacodynamics. CTD is known to have a longer duration of action and a more potent diuretic effect, which could potentially lead to a greater likelihood of electrolyte disturbances and renal impairment. On the other hand, HCTZ, while less potent, may offer a more favorable safety profile in these particular aspects due to its shorter duration of action. Our results reveal increased risks of hypokalemia and hyponatremia with the use of CTD compared with HCTZ, and these electrolyte imbalances can lead to potentially fatal clinical complications if left untreated. However, given the lack of available evidence, these findings can still not be considered confirmatory and it is imperative to evaluate these further. Moreover, given the shorter duration of action of HCTZ, it is also important to investigate the long-term effects and safety of administering HCTZ compared with CTD in patients with HTN.

Meanwhile, our results with respect to AKI depicted conflicting findings on sensitivity analyses and demonstrated the potential benefit of HCTZ on AKI compared with CTD. Cardiorenal interactions are often the basis of life-threatening complications in patients with cardiovascular diseases such as HTN. Treatments providing any sort of disease modification that concurrently disseminate potential renal safety in patients with HTN can alleviate clinical outcomes and may prolong survival as well. Future research should focus on confirming the renal safety of these thiazide diuretics in patients with HTN. Such ambiguous findings call for clinicians to consider patient comorbidities, drug–drug interactions, and the overall patient profile when administering CTD.

While recent evidence from the 2020 part D Medicare expenditures demonstrates that HCTZ was prescribed almost eight times more than CTD, it is important to understand that CTD bears similar clinical efficacy and in light of further research, it may be prescribed keeping in mind the patient’s clinical picture[16]. For example, future research evaluating the dose-dependent efficacy and safety of CTD vs HCTZ may propose additional ways of tailoring patient management. Moderate to high heterogeneity in some analyses may reflect clinical and methodological differences across the included studies. These may include variability in patient populations (e.g., baseline cardiovascular or renal risk), differences in outcome definitions (particularly for endpoints like AKI or hospitalization), and inconsistencies in treatment protocols such as diuretic dosing, duration of follow-up, and concomitant medications. These findings have potential implications for clinical decision-making, particularly in selecting a thiazide diuretic for patients with HTN who are at risk of cardiovascular or renal events. The observed differences in efficacy and safety outcomes may support a preference for chlorthalidone over HCTZ in specific clinical contexts.

Our meta-analysis provides the most updated findings with respect to the comparative efficacy and safety of CTD and HCTZ. We assess the comparative effect of CTD vs HCTZ on not only MACE but its individual components as well. However, the limitations of our study must be acknowledged. We could not evaluate individual patient level data and the inherent biases present in each individual study could not be accounted for. Additionally, the inclusion of Neutel *et al*, where the comparison involved combination therapies (e.g., azilsartan medoxomil/chlorthalidone versus olmesartan medoxomil/HCTZ), introduces a potential confounder. While both second drugs in these combination arms were ARBs, leading us to assume that baseline medications of the same class might not significantly affect the individual responses of the thiazide diuretics, this remains an assumption and could influence the overall findings. Dose-related differences in efficacy and safety between HCTZ and CTD were not consistently reported across studies and may partly explain variability in outcomes. Future research should explore dose–response relationships in head-to-head trials. Additionally, due to the sparsity of available evidence, only four studies could be included in our meta-analysis. This could lead to limited generalizability of this analysis. Moreover, two of our included studies were double-arm observational studies and not randomized clinical trials, which could have led to some confounding bias in our results. Lastly, the risk of bias in the two included randomized controlled trials was mostly unclear, particularly due to limited information on randomization, allocation concealment, and blinding procedures.

As a result, future clinical trials are needed to confirm our findings. Additionally, further research is warranted to explore the differences in safety outcomes between CTD and HCTZ in order to identify the subgroups of patients who may benefit more from one diuretic over the other. Determining such high-risk patients is essential to accordingly prescribe them HCTZ or safe doses of CTD.

## Conclusion

In conclusion, CTD and HCTZ demonstrate similar efficacy profiles with respect to cardiovascular outcomes in patients with HTN. However, HCTZ appears to be a safer option due to its lower risk of precipitating electrolyte abnormalities like hypokalemia and hyponatremia. Meanwhile, the risk of AKI remains ambiguous and needs to be elucidated by future research. These findings bear important implications for the management of HTN and can aid clinicians in making informed treatment choices for their patients.

## Data Availability

All data analyzed here have been sourced from already published studies, and as such can be obtained from those studies accordingly.

## Research registration unique identifying number (UIN)

1. Name: Institute for Health Research (NIHR) International prospective register of systematic reviews (PROSPERO) 2. Unique Identifying number or registration ID: CRD42025632848 3. Hyperlink to your specific registration (must be publicly accessible and will be checked): https://www.crd.york.ac.uk/PROSPERO/display_record.php?RecordID=632848.
